# Facile and Green Synthesis of Highly Fluorescent Carbon Quantum Dots from Water Hyacinth for the Detection of Ferric Iron and Cellular Imaging

**DOI:** 10.3390/nano12091528

**Published:** 2022-05-01

**Authors:** Pei Zhao, Qin Zhang, Juanjuan Cao, Cheng Qian, Jing Ye, Siyuan Xu, Yonggui Zhang, Yanbin Li

**Affiliations:** 1 College of Biological and Food Engineering, Anhui Polytechnic University, Wuhu 241000, China; lvxmzp@163.com (P.Z.); cjj18890973009@163.com (J.C.); qiancheng9981@163.com (C.Q.); a2235672022@163.com (J.Y.); shanyazi0605@163.com (S.X.); xjagcdzyg@163.com (Y.Z.); 2 Anhui Engineering Laboratory for Industrial Microbiology Molecular Breeding, College of Biological and Food Engineering, Anhui Polytechnic University, Wuhu 241000, China

**Keywords:** water hyacinth, green synthesis, carbon quantum dots, Fe^3+^ sensing, fluorescent probe, cellular imaging

## Abstract

Natural biomass is used for facile synthesis of carbon quantum dots (CQDs) with high fluorescence, owing to its abundance, low cost, and eco-friendliness. In this study, a bottom-up hydrothermal method was used to prepare CQDs from water hyacinth (wh) at a constant temperature of 180 °C for 12 h. The synthesized wh-CQDs had uniform size, amorphous graphite structure, high water solubility (containing multiple hydroxyl and carboxyl groups on the surface), excitation light-dependent characteristics, and high photostability. The results showed that the aqueous solution of CQDs could detect Fe^3+^ rapidly, sensitively, and highly selectively with a detection limit of 0.084 μM in the linear range of 0–330 μM, which is much lower than the detection limit of 0.77 μM specified by the World Health Organization. More importantly, because the wh-CQDs were synthesized without any additives, they exhibited low toxicity to *Klebsiella* sp. cells even at high concentrations. Moreover, wh-CQDs emitted bright blue fluorescence in *Klebsiella* sp. cells, indicating its strong penetrating ability. Correspondingly, the fluorescent cell sorting results also revealed that the proportion of cell internalization reached 41.78%. In this study, wh-CQDs derived from natural biomass were used as high-performance fluorescent probes for Fe^3+^ detection and *Klebsiella* sp. imaging. This study is expected to have great significance for the application of biomass carbon spots in the field of cellular imaging and biology.

## 1. Introduction

Biomass materials are a crucial renewable source of carbon on earth. Based on differences in structure, composition, and molecular weight, raw materials for synthetic biomass can be divided into three categories: micro-molecules derived from biomass (e.g., citric acid, glucose, etc.), biomass components (e.g., cellulose, hemicellulose, lignin, etc.), and natural biomass (e.g., straw, crab shells, etc.) [1,2]. Approximately 181 billion tons of biomass waste is annually produced and not treated effectively worldwide [3]. This not only burdens the ecology but also is a waste of a significant amount of resources. Currently, many researchers use forest by-products, agricultural residues, food waste, and industrial residues, such as lignin, chitosan, rice husk, cherry stone powder, waste tar, as green raw materials for synthetic carbon nanoparticles [4,5,6,7,8]. The effective conversion of biomass waste into carbon nanoparticles could reduce the harm of organic waste to the environment through resource management and alleviate the pressure of resource shortage.

Quantum dots are semiconductor nanoparticles, and traditional fluorescent quantum dots are semiconductor quantum dots (SQDs), which emit light at a specific frequency under a certain electric field or optical pressure and are used in the fields of sensing [9], drug carriers [10], and biological imaging [11]. However, the applications of SQDs in analytical detection and biological imaging are limited because they contain heavy metal ions and are highly toxic. Carbon quantum dots (CQDs) are a member of the carbon nanomaterial family; they are generally smaller than 10 nm and are mainly composed of C and O [12]. CQDs have better biocompatibility, low toxicity, water solubility, and photoluminescence characteristics than SQDs and can be applied in the fields of bioimaging, drug carriers, gene delivery, metal ion detection, sensing and nanothermometer [13,14,15,16,17,18]. The synthesis methods of CQDs include top-down and bottom-up methods: cracking of different carbon source materials and carbonization of small molecules or polymers [19]. Among them, the hydrothermal method, a bottom-up approach possessing the advantages of energy efficiency, low cost, and convenient operation, is mainly used to carbonize organic matter to form luminescent CQDs at high temperatures and pressures. Trace elements and heteroatoms present in the biomass accumulate on the surface to form abundant functional groups, which are good precursors for the formation of CQDs [20]. Biomass, such as saponins, coconut husk, avocados, corncobs, kiwifruit, passion fruit peel, and corn stalks, can be used as carbon precursors in the green synthesis of CQDs and show great potential for CQDs production [21,22,23,24,25,26,27]. Moreover, biomass CQDs possess advantages in the field of bioimaging because of their excellent fluorescence properties and low cytotoxicity. Chan et al. reviewed the synthesis of CQDs from various natural biomass [28]. However, the current objects of biomass CQD imaging studies are primarily cancer cells and seldom bacterial cells (e.g., cells of *Escherichia* *coli* and *Staphylococcus aureus*) [29,30,31,32]. To the best of our knowledge, imaging and toxicity studies of biomass CQDs on the hydrogen-producing bacterium *Klebsiella* sp. have rarely been reported. Therefore, it is of vital importance to use biomass CQDs with low toxicity obtained via green synthesis to trace bacterial growth behaviors during hydrogen production. 

Iron is one of the most important trace elements in biological systems and a common heavy metal pollutant in industrial wastewater and drinking water [33]. The detection of ferric iron is of great importance for biological systems, as it plays a crucial role in the biochemical pathways of living cells [34]. Excess or lack of Fe^3+^ ions may cause body disorders and various diseases, where excess Fe^3+^ ions can deteriorate the function of heart, pancreas, and lungs, and even lead to cancer, whereas a lack of Fe^3+^ leads to anemia [35]. Therefore, it is essential to develop a safe, efficient, sensitive, and selective method for detecting Fe^3+^. Among many methods, the detection of Fe^3+^ by biomass-derived CQDs has the advantages of high sensitivity, low detection limit, simple operation, and fast response speed compared with spectrophotometry, colorimetry, and inductively coupled methods with complex operation and limited accuracy. For example, Chen et al. used lignin as a precursor to synthesize CQDs with a higher quantum yield (QY), wider detection range, and lower detection limit than some reported CQDs [36]. Therefore, excellent selectivity and sensitivity indicate that biomass CQDs are promising probes for detecting Fe^3+^.

In this study, biomass CQDs with blue fluorescence properties were synthesized by hydrothermal green metho using biomass water hyacinth (wh) as a carbon source, with a QY of 3.3%. Compared with the reported carbon dots derived from wh [37], the synthesis in the present study was greener, the particle size was more uniform, and the product could be more widely used in biological applications because no chemical reagents were added. The physicochemical properties of wh-CQDs, such as particle size, crystal structure, surface functional groups, elemental composition, and optical properties, were characterized using various techniques. The wh-CQDs were tested for Fe^3+^, and their detection range for Fe^3+^ was 0–330 μM; simultaneously, their detection limit was 0.084 μM, which is much lower than the detection limit specified internationally. Finally, the biocompatibility of the biomass-derived wh-CQDs was evaluated by cellular imaging, toxicity studies, and flow cytometry analysis on *Klebsiella* sp. The experiments revealed that our synthesized wh-CQDs could be internalized into *Klebsiella* sp. and showed blue fluorescence, indicating good biocompatibility. The activity of *Klebsiella* sp. remained above 90% at 1000 μg/mL of wh-CQDs. These results suggest that biomass wh-CQDs have good potential for biomedical applications.

## 2. Materials and Methods

### 2.1. Materials

Wh was collected locally, washed thrice with water, and air-dried. The samples were crushed to obtain a fine powder and sieved (Joyoung, Wuhu, China). Microporous membrane (0.22 μm), peptone, yeast extract, and agar were purchased from Sangon Biotech (Shanghai, China). Quinine sulfate (98%, suitable for fluorescence) and other chemical reagents, such as NaOH, NaCl, and the metal ion compounds, were of analytical reagent grade, supplied by Macklin Biotech (Shanghai, China), and used without further purification. Ultrapure water (HHitech, Shanghai, China) was used for all experiments.

### 2.2. Preparation of wh-CQDs

**Figure 1:**
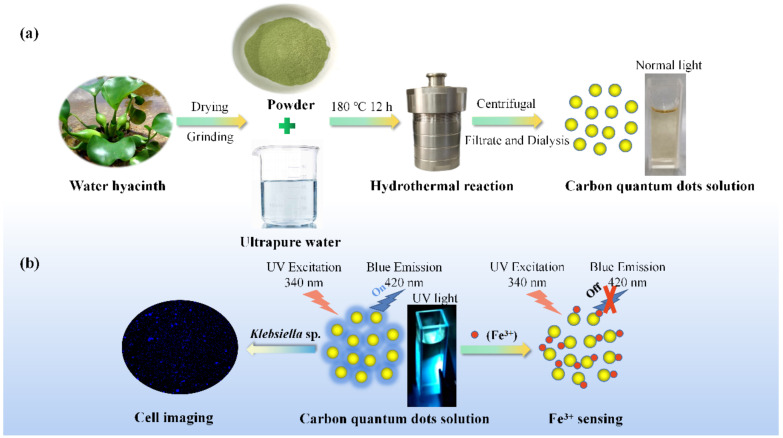
(**a**) Schematic of CQDs derived from wh and (**b**) their applications.

Synthesis of wh-CQDs: According to a published process, wh-CQDs were synthesized using a one-step hydrothermal method with some modifications (Figure 1) [37]. In this typical synthesis process, approximately 5 g of wh powder was dispersed in 50 mL of purified water under stirring, after which the mixture was transferred into a 100 mL Teflon-lined stainless-steel autoclave and heated at 180 °C for 12 h. After cooling to room temperature, the mixture was centrifuged at 8000 rpm for 20 min, and the supernatant was filtered using a 0.22 μm microporous membrane to remove insoluble precipitate. The supernatant was then subjected to dialysis against purified water with a dialysis bag (molecular weight cutoff: 1 kDa) for 24 h to remove small molecules. The brown solution of wh-CQDs was stored at 4 °C for characterization and further use.

### 2.3. Characterizations

The surface morphology of the wh-CQDs was determined using a Tecnai G2 F30 S-TWIN field-emission transmission electron microscopy (TEM and HRTEM, FEI Corp., Portland, OR, USA) at an accelerating voltage of 200 kV. The particle size of the wh-CQDs was analyzed using image processing software Image J 1.52 and TEM micrographs, and the size distribution was calculated using Origin 8.0. Powder X-ray diffraction (XRD) analysis was conducted using a Bruker D8 Advance diffractometer (Bruker Corp., Billerica, MA, USA) with Cu Kα radiation and a scanning scope of 2θ = 10–90°. The functional group information of the wh-CQDs was analyzed using Fourier transform infrared spectroscopy (FTIR, Nicolet IS10, Nicolet Corp., Madison, WI, USA) in the range 400–4000 cm^−1^. For each measurement, the samples were scanned 64 times at a resolution of 4 cm^−1^. X-ray photoelectron spectroscopy (XPS) was used to characterize the content and robustness of various elements in the samples (Thermo, Waltham, MA, USA). Ultraviolet-visible (UV-Vis) absorption spectra were obtained using a Shimadzu 2550 UV-Vis spectrophotometer (Shimadzu, Tokyo, Japan). The two- and three-dimensional fluorescence spectra and fluorescence intensities of the wh-CQDs were recorded using an F-7100 fluorescence spectrometer (Hitachi, Tokyo, Japan) with the excitation and emission slits of 5 nm. Fluorescence lifetime measurements were performed on an FluoroMax-4 time-resolved fluorescence spectrophotometer (HORIBA, Kyoto, Japan).

### 2.4. QY Calculations

QY calculations were performed using an established method. Quinine sulfate solution (Φ = 54% at 360 nm excitation light, η = 1.33) was used as a standard reference to calculate the relative QY of the wh-CQDs. The specific operation steps were as follows: five different concentrations of wh-CQDs dispersions in water and quinine sulfate solutions in 0.1 M H_2_SO_4_ were prepared. The absorbance of both wh-CQDs and quinine sulfate solution was adjusted to below 0.1 to reduce the internal filtration effect. The QY of wh-CQDs was calculated using the following Equation (1) [38]:

(1) $${\;\Phi }_{S}=\Phi_{R}\left(\frac{{\mathrm{Grad}}_{S}}{{\mathrm{Grad}}_{R}}\right)\left(\frac{\eta_{S}{}^{2}}{\eta_{R}{}^{2}}\right)$$

where Grad_S_ and Grad_R_ are the slopes of the integrated areas of the fluorescence intensity, and absorbance for wh-CQDs and quinine sulfate, respectively, and η is the refractive index of the solvent; subscripts S and R correspond to the wh-CQDs and quinine sulfate, respectively.

### 2.5. Stability of wh-CQDs

The effects of pH (2–12), salt ion concentration (0.02, 0.04, 0.06, 0.08, and 0.1 M), and UV irradiation time (0, 10, 20, 30, 40, 50, and 60 min) on the fluorescence intensity of wh-CQDs were assessed. The fluorescence intensity was recorded using a spectrofluorometer at an excitation wavelength of 340 nm.

### 2.6. Sensitivity and Selective Detection of Fe^3+^ Ions

For the sensitivity experiment, ferric chloride hexahydrate (FeCl_3_) was dissolved in ultrapure water to prepare solutions with different Fe^3+^ concentrations. A 300 μL solution of wh-CQDs (1 mg/mL) was diluted 10-fold with ultrapure water to detect Fe^3+^; 3 mL of wh-CQDs (100 μg/mL) was mixed with 100 μL of Fe^3+^ solutions of different concentrations, and the mixtures were maintained for 3 min at room temperature. Fluorescence intensity was recorded using a spectrofluorometer at 340 nm with excitation and emission slits of 5 nm at a PMT voltage of 600 V. 

To compare the sensitivity of the wh-CQDs toward different ions, the fluorescence spectra of different metal ions (Fe^2+^, Mg^2+^, NH_4_^+^, Ni^2+^, Zn^2+^, K^+^, Na^+^, Mn^2+^, Co^2+^, Ba^2+^, Al^3+^, Ca^2+^, and Cu^2+^, 10 mM) with CQDs were obtained according to the steps described above.

### 2.7. Cell Viability Assay

The viability of *Klebsiella* sp. was determined using 96-well plates. First, 10^5^ colony-forming units (CFUs) of cells were obtained by gradient dilution of optical density (OD). Then, 100 μL of 10^5^ CFU of bacterial cells was mixed with 100 μL of wh-CQDs of different concentrations (31.25, 62.5, 125, 250, 500, 1000, and 2000 μg/mL) prepared in phosphate-buffered saline (PBS); bacterial culture without wh-CQDs was used as a control. After incubation at 37 °C for 24 and 48 h, the absorbance was recorded at a wavelength of 600 nm. Cell viability was calculated using Equation (2) [39]:

(2) $$\;\mathrm{Cell}\;\mathrm{viability}\;\left(\%\right)=\frac{{\mathrm{OD}}_{\mathrm{treated}}}{{\mathrm{OD}}_{\mathrm{control}}}\times 100\%\;$$

where OD_control_ is the OD in the absence of wh-CQDs and OD_treated_ is the OD in the presence of wh-CQDs.

### 2.8. Cell Imaging

*Klebsiella* sp. were streaked on LB solid plates and cultured at 37 °C for 24 h. A single colony was inoculated in LB liquid medium, and an appropriate amount of synthetic CQD solution was added to the medium (150 μL of 500 μg/mL wh-CQDs in 2 mL medium). The mixture was incubated overnight at 37 °C under shaking at 200 rpm. The next day, 1 mL of culture medium was centrifuged and washed three times with PBS. Then, the bacterial cells were suspended in 500 μL of PBS and kept at 4 °C for fluorescence imaging using Leica confocal microscope.

### 2.9. Flow Cytometry Analysis of Intracellular wh-CQDs

Flow cytometric analysis of *Klebsiella* sp. cells incubated with wh-CQDs was performed using a Beckman Coulter flow cytometer. Briefly, *Klebsiella* sp. bacterial cells were incubated with 500 μg/mL wh-CQDs for 12 and 24 h at 37 °C to obtain 10^5^ CFU/mL of bacteria by adjusting the OD value; 1 mL of the culture medium was centrifuged and washed three times with PBS, and then the bacterial cells were suspended in 500 μL of PBS for flow cytometric analysis, with the control without wh-CQDs. Flow cytometry data were analyzed using CytExpert software.

## 3. Results and Discussion

### 3.1. Synthesis and Characterization of wh-CQDs

Wh is an outstanding precursor for synthetic carbon nanodots because it contains many secondary metabolites, such as phenols, flavonoids, sterols, terpenoids, and anthraquinones, which act as stabilizers and accelerators in the green synthesis of nanoparticles [40,41]. In wh-CQDs synthesis, the hydrolysis and decomposition of carbohydrates in wh through a bottom-up hydrothermal method leads to the loss of water, and the formed products react to form aromatic compounds during polymerization and condensation. Finally, a nuclear explosion occurs to form carbon quantum dots. Green synthesis enables the production of carbon and carbon hybrid materials with controllable structure and morphology in an energy-saving manner [42,43]. The synthetic route for the CQDs derived from wh is illustrated in Figure 1.

**Figure 2:**
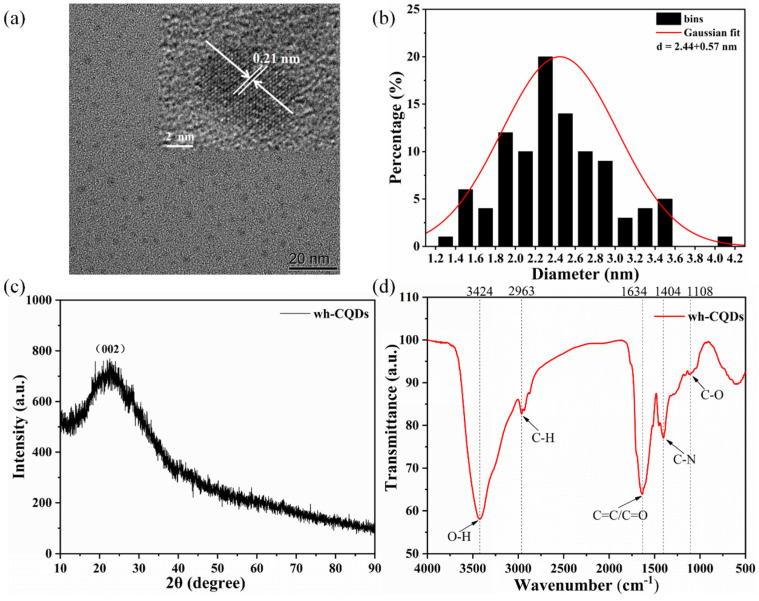
Characterization of wh-CQDs: (**a**) TEM image (inset: HRTEM image), (**b**) the size distribution of particles, (**c**) XRD pattern, (**d**) FTIR spectra.

The morphology of the wh-CQDs was evaluated using TEM. As shown in Figure 2a, the as-prepared wh-CQDs were uniform in size and well-dispersed throughout the cross-section with high monodispersity. As shown in the inset of Figure 2a, the HRTEM image showed that the wh-CQDs exhibited an interplanar spacing of 0.21 nm, which confirmed the presence of graphitic carbon structures [44]. In addition, the corresponding particle size histograms of the wh-CQDs were obtained by selecting 100 nanoparticles for analysis using the ImageJ 1.52 software (Figure 2b). The statistical results showed that the particle size distribution of wh-CQDs ranged from 1.2 to 4.2 nm with an average diameter of 2.44 ± 0.57 nm. The small particle size and high dispersibility of the synthesized wh-CQDs are attributable to the uniform energy and high pressure generated in the hydrothermal reaction. 

Figure 2c shows the XRD patterns of the synthesized wh-CQDs recorded in the range of 2θ = 10–90°. The XRD pattern of the wh-CQDs exhibits a broad peak at 2θ = 23°, which was consistant with the standard XRD pattern of PDF #39-1889 card and could be assigned to the diffraction of the (002) lattice plane, confirming the disordered graphitic structure [45,46]. Therefore, the wh-CQDs were amorphous spherical nanoparticles based on the TEM and XRD results. Furthermore, the surface structure and functional groups of the wh-CQDs were characterized using FTIR spectroscopy (Figure 2d). The FTIR spectra showed different characteristic absorption peaks attributed to the stretching and bending vibrations of different groups (O–H, C–H, C=O, C=C, C–N, and C–O). The broad band at 3424 cm^−1^ was attributed to the O–H stretching vibration, and the absorption band at 2963 cm^−1^ was assigned to C–H stretching [47]. The absorption peaks at 1634, 1404, 1108 cm^−1^ were assigned to the stretching vibrations of C=O/C=C, C–N, and C–O groups, respectively [48,49]. These functional groups demonstrate that the surface of wh-CQDs contained hydroxyl and carboxylic groups, which determine the hydrophilicity and stability of wh-CQDs in aqueous solutions.

**Figure 3:**
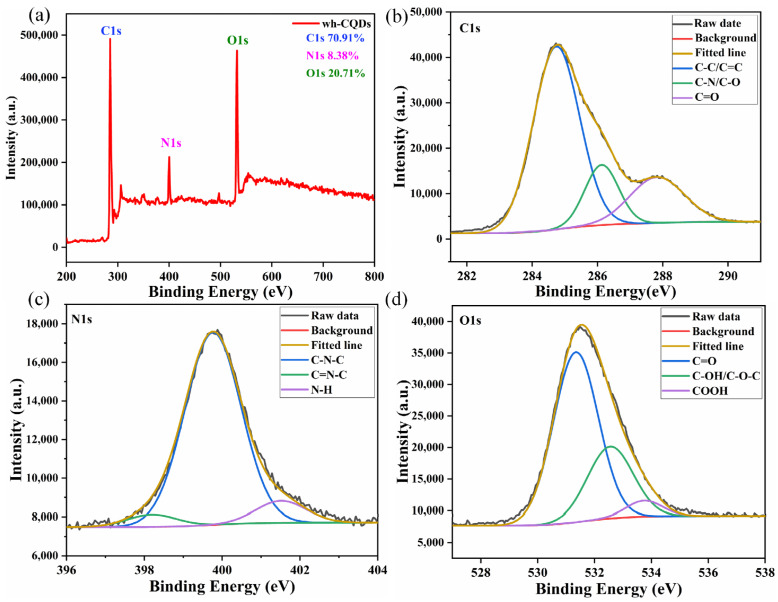
(**a**) XPS full-survey spectrum of wh-CQDs. The high-resolution deconvoluted XPS peaks of wh-CQDs for (**b**) C1s, (**c**) N1s, and (**d**) O1s.

To further determine the elemental composition of the wh-CQDs, the samples were characterized using XPS. As shown in Figure 3a, the XPS spectra of wh-CQDs obtained in the full wavelength range exhibit three typical strong peaks at 284.8, 399.8, and 531.6 eV, which were attributed to C1s, N1s, and O1s, respectively, indicating that the wh-CQDs contained C (70.91%), N (8.38%), and O (20.71%). The high-resolution C1s spectrum (Figure 3b) reveals three main deconvolution peaks at 284.7, 286.1, and 287.8 eV, corresponding to the C–C/C=C, C–N/C–O, and C=O surface groups of the wh-CQDs [12]. Similarly, the three peaks in the N1s spectrum (Figure 3c) appeared at 399.8, 398.2, and 401.5 eV, representing C–N–C, C=N–C, and N–H, respectively [50,51]. In the O1s spectrum (Figure 3d), three main peaks with binding energies of 531.3, 532.6, and 533.8 eV were observed; they were assigned to C=O, C–OH/C–O–C, and –COOH, respectively [35]. In general, XPS analysis showed that the surface of wh-CQDs contained multiple O- with N- functional groups, which was consistent with the FTIR results and illustrated that wh-CQDs are highly water-soluble.

### 3.2. Optical Properties of wh-CQDs

**Figure 4:**
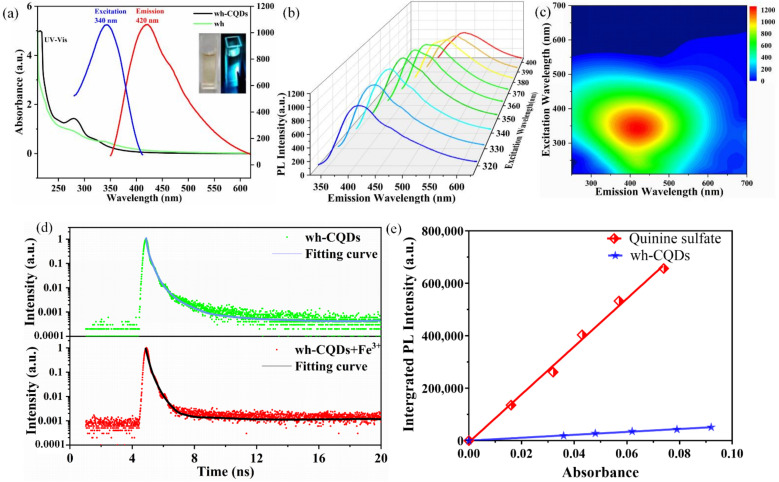
(**a**) UV-vis absorption with maximum fluorescence excitation and fluorescence emission spectra of the wh-CQDs, (**b**) Fluorescence emission spectra of the wh-CQDs at different excitation wavelengths ranging from 320 to 400 nm (increments of 10 nm), (**c**) 3D fluorescence contour map of the wh-CQDs, (**d**) Fluorescence decay curve of wh-CQDs before and after the addition of 100 μM of Fe^3+^, (**e**)QY measurement of wh-CQDs using quinine sulfate as the reference.

The optical characteristics of the wh-CQDs (100 μg/mL) were determined by measuring the UV-Vis absorption spectra, photoluminescence spectra and time-resolved fluorescence spectra. The diluted wh-CQDs exhibited good water solubility at room temperature. The aqueous wh-CQD solution was brown under daylight and bright blue under 365-nm UV light (inset, Figure 4a). Figure 4a shows the ultraviolet absorption and fluorescence spectra of the wh-CQDs. The wh powder has no absorption peak at 270 nm. Compared with the wh powder, the wh-CQDs exhibited a large absorption peak at 270 nm, which was attributed to the C=C/C=N π–π* transition of the SP^2^ hybrid domain. In the fluorescence spectra of wh-CQDs, the optimal excitation wavelength was 340 nm, and the optimal emission wavelength was 420 nm. As shown in Figure 4b, the fluorescence intensity of the wh-CQDs increased at excitation wavelengths of 320–340 nm and decreased at 340–400 nm. Wh-CQDs exhibited typical excitation-light-dependent emission characteristics. There are two typical luminescence models for this property: bandgap transitions in the conjugated π domain and surface defects of CQDs. According to the former mechanism, some studies have attributed the luminescence redshift of CQDs to the quantum size effect [52,53]. In the latter mechanism, the surface state is the main factor affecting the fluorescence change [54,55]. In the present study, owing to the size composite Gaussian distribution of the wh-CQDs, the excitation light dependence may be caused by surface-state defects. Figure 4c shows the three-dimensional fluorescence spectra of the wh-CQDs with a large fluorescence circle at an excitation wavelength of 340 nm and an emission wavelength of 420 nm, corresponding to the fluorescence emission spectra of the wh-CQDs. Figure 4d shows the fluorescence decay profile for wh-CQDs at an excitation/emission wavelength of 340/420 nm, which is in line with biexponential fitting curve with an average lifetime of 2.2 ns. The QY of the wh-CQDs was calculated to be 3.3%, using quinine sulfate as the fluorescence standard reference (Figure 4e). As shown in Table S1, five different concentrations of wh-CQDs and quinine sulfate solutions were explained.

### 3.3. Stability of wh-CQDs

**Figure 5:**
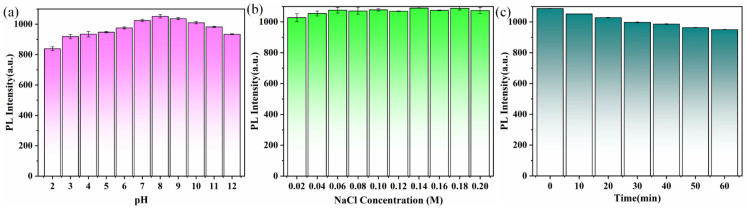
(**a**) Stability of fluorescence intensity of the wh-CQDs at different pH values, (**b**) NaCl concentrations, (**c**) exposure to UV light (0–60 min).

To determine the stability of the wh-CQDs in cell imaging and sensing, the effects of acidic/basic, salt ionic concentrations, and irradiation times on the fluorescence intensity was examined. As shown in Figure 5a, the fluorescence intensity of the wh-CQDs was highest at pH 8 and tended to be stable in weakly acidic and weakly alkaline pH environments. No apparent changes were observed in the fluorescence intensity of the wh-CQDs at NaCl concentrations of 0.02–0.2 M (Figure 5b). In addition, the fluorescence intensity of the wh-CQDs remained stable under uninterrupted UV irradiation (Figure 5c). All experiments were based on the results of three parallel experiments. These results are consistent with those of previous studies [56,57]. The outstanding stability of wh-CQDs enables their further application in sensing and bioimaging.

### 3.4. Fluorescent and Colorimetric Detection of Fe^3+^

**Figure 6:**
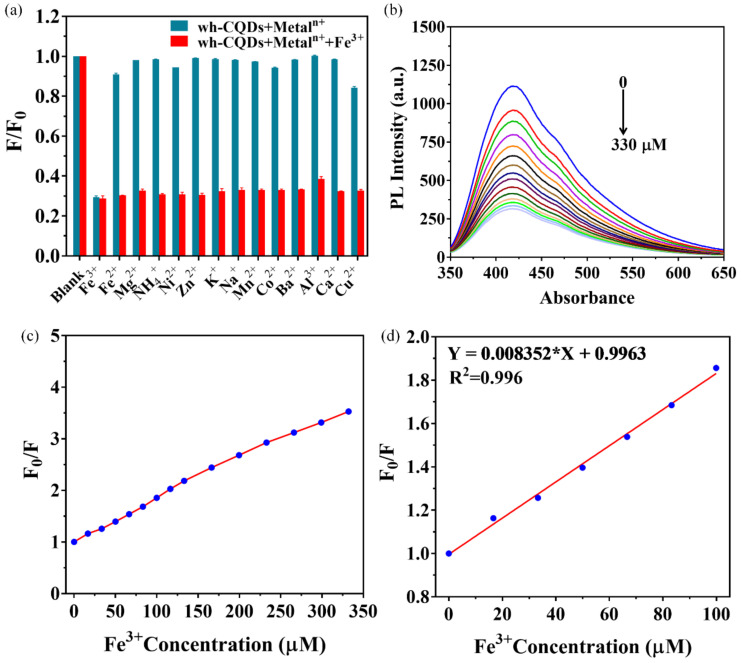
(**a**) The relative fluorescence intensities (F/F_0_) of the wh-CQDs in the presence of individual metal ions (blue column) and co-presence of Fe^3+^ ion with other metal ions (red column), (**b**) the fluorescent emission spectra of the wh-CQDs under various Fe^3+^ ion concentrations (0–330 μM), (**c**) the dependence of relative fluorescence (F/F_0_) of the wh-CQDs on the concentration of Fe^3+^ ions ranging from 0–330 μM, (**d**) the Stern–Volmer plots for the concentration of Fe^3+^ in the range of 0–100 μM.

The applications of wh-CQDs in the environmental sciences and biology have been extensively investigated owing to their strong fluorescence properties and excellent stability. In this study, we used wh-CQDs as fluorescent probes to detect Fe^3+^. The fluorescence intensity of the wh-CQDs was recorded at an excitation wavelength of 340 nm. The selectivity of wh-CQDs for Fe^3+^ was evaluated by adding metal ions in the presence and absence of Fe^3+^, including Fe^2+^, Mg^2+^, NH_4_^+^, Ni^2+^, Zn^2+^, K^+^, Na^+^, Mn^2+^, Co^2+^, Ba^2+^, Al^3+^, Ca^2+^, and Cu^2+^ (Figure S1). As shown in Figure 6a, F and F_0_ are the fluorescence intensities at 340 nm in the presence and absence of the metal ions, respectively. Apparently, the fluorescence of wh-CQDs was quenched by 70% when Fe^3+^ was introduced, while in the presence of other metal ions the change was small (Fe^2+^, Ni^2+^, Co^2+^, and Cu^2+^) or negligible (Mg^2+^, NH_4_^+^, Zn^2+^, and Al^3+^). At the same time, when Fe^3+^ was introduced into the wh-CQD solution in the presence of metal ions, the fluorescence intensity was still significantly quenched (red column). Therefore, the as-prepared wh-CQDs exhibited fascinating anti-interference ability with high selectivity for Fe^3+^ compared to other metal ions. The high selectivity of the wh-CQDs can be attributed to the chelation interactions between the N and O functional groups on the surface of the wh-CQDs and Fe^3+^. Specifically, the electron-deficient Fe^3+^ maintained a half-filled 3d orbital to accept electrons, and the −OH and −COOH groups on the surface of the wh-CQDs after the introduction of Fe^3+^ rapidly reacted with Fe^3+^ to promote the transfer of excited electrons from the wh-CQDs to the 3d orbital of Fe^3+^, resulting in non-radiative electron/hole recombination that quenched the fluorescence of the wh-CQDs [58,59,60]. The quenching mechanism of wh-CQDs induced by Fe^3+^ was investigated by measuring decay lifetimes. The decay lifetime would be a strong evidence to differentiate static quenching (decay lifetime hardly changed) and dynamic quenching (decay lifetime changed) [61,62,63]. The fluorescence lifetime of wh-CQDs is calculated as 2.2 ns in the absence of Fe^3+^, and the change in fluorescence lifetime after the addition of Fe^3+^ was almost negligible as 1.9 ns (Figure 4d). Therefore, static quenching plays a major role in the fluorescence quenching of wh-CQDs.

For the sensitivity experiments, the fluorescence intensities at different concentrations of Fe^3+^ were investigated under 340-nm excitation light (Figure 6b). The fluorescence intensity of the wh-CQDs was sharply quenched by adding Fe^3+^ from 0 to 330 μM (Figure 6c). The ratio of the initial fluorescence intensity to the fluorescence intensity (F_0_/F) at 340 nm excitation light was a linear function of the Fe^3+^ concentration in the range of 0–100 μM with a correlation coefficient of 0.996, as shown in Figure 6d. The fluorescence quenching of the wh-CQDs follows the Stern-Volmer Equation (3).

(3) $$\frac{F_{0}}{F}-1=K_{SV}\left[C\right]$$

where F_0_ and F are the emission intensities of the wh-CQDs at 340 nm in the absence and presence of Fe^3+^, respectively; K_sv_ is the Stern–Volmer burst constant; and [C] is the Fe^3+^ concentration. The detection limit was calculated using the formula LOD = 3σ/S, where σ represents the standard deviation of the fluorescence intensity of the blank wh-CQDs, and S is the slope of the linear function (n = 10). From the results obtained, we calculated the detection limit to be 0.084 μM. Notably, the obtained detection limit of Fe^3+^ by the wh-CQDs was much lower than that proposed by the World Health Organization (5.36 μM), indicating that the wh-CQDs can be applied in highly selective sensing of Fe^3+^. A comparison of different systems for Fe^3+^ determination is summarized in Table 1, indicating that the detection limits of CQDs derived from biomass are comparable to or lower than those proposed earlier.

### 3.5. Cytotoxicity and Cell Imaging

**Figure 7:**
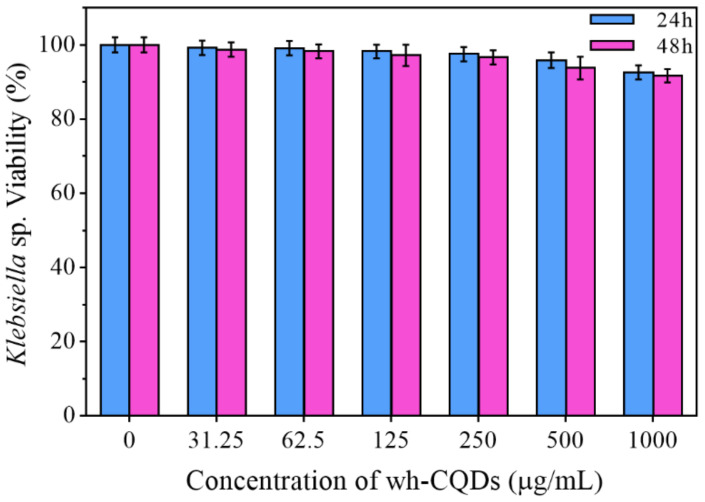
Cell viability assays of the *Klebsiella* sp. treated with different concentrations of wh-CQDs for 24 and 48 h.

CQDs are used as probes in various fields owing to their excellent tunable fluorescence properties, colorful fluorescence emission, small size, high photostability, and biocompatibility [64,65,66]. In the present study, to assess the cell viability of wh-derived biomass carbon spots, hydrogen-producing bacterium *Klebsiella* sp. was used as a model for toxicity studies. As shown in Figure 7, cell viability was unaffected by changes in the concentration of the wh-CQDs. At the wh-CQD concentration of 1000 μg/mL, cell viability was still above 90%. At low concentrations, the cell viability was largely unaltered, indicating that wh-CQDs possessed low toxicity toward the hydrogen-producing bacterium *Klebsiella* sp.

**Figure 8:**
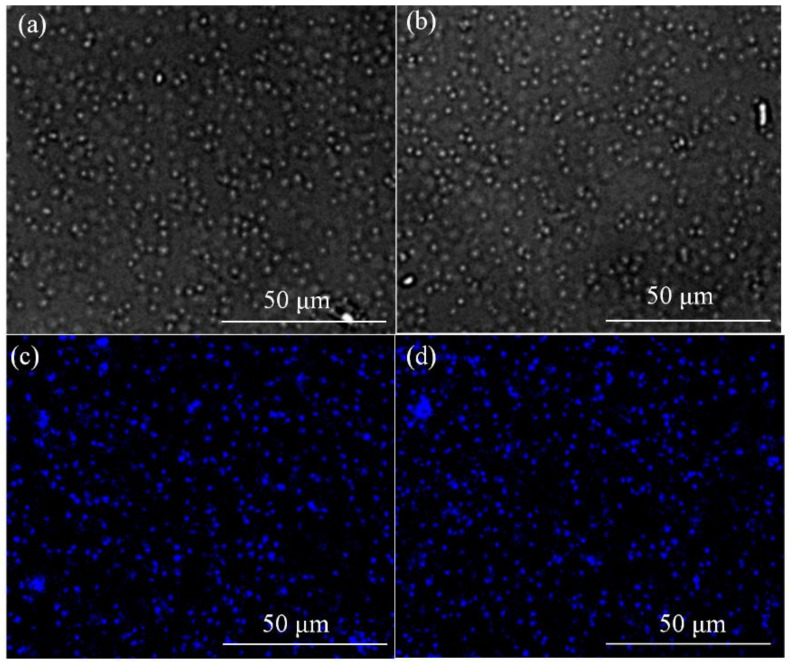
Fluorescence image of *Klebsiella* sp. cells incubated with wh-CQDs (500 μg/mL) for 12 h under (**a**–**b**) bright field and (**c**–**d**) excitation wavelength of 375–407 nm.

As shown in Figure 8, imaging studies were performed after culturing the hydrogen-producing bacterium *Klebsiella* sp. in a medium containing 500 μg/mL wh-CQDs for 12 h. As observed using laser confocal microscopy, the CQDs exhibited an intense blue fluorescence signal at excitation wavelengths of 375–407 nm with outstanding imaging potential. Notably, *Klebsiella* sp. cell walls contain various polysaccharides and lipids, which make them difficult to be labeled. The wh-CQDs could enter the bacterial membrane via endocytosis, indicating that the wh-CQDs have strong penetrating ability. Therefore, owing to the strong fluorescence, stability, and good biocompatibility of wh-CQDs, they are expected to be an effective fluorescent probe for bacterial cell tracers in the bioenergy field.

### 3.6. Flow Cytometry Analysis of Intracellular wh-CQDs

**Figure 9:**
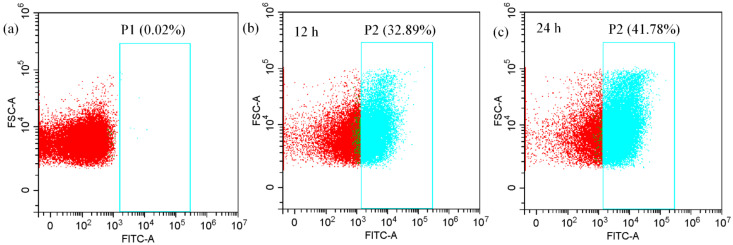
Proportion of wh-CQDs internalized in *Klebsiella* sp. by flow cytometry analyses. (**a**) without wh-CQDs, (**b**) *Klebsiella* sp. cells incubated with wh-CQDs (500 mg/mL) for 12 h and (**c**) 24 h.

Based on the cellular imaging results, we determined the proportion of wh-CQD uptake by *Klebsiella* sp. cells. To this end, cells were incubated with 500 μg/mL wh-CQDs and analyzed by flow cytometry after incubation for 12 and 24 h at 37 °C. The emission of wh-CQDs was analyzed in the FITC fluorescence channel. As shown in Figure 9a, the relative fluorescence intensity of 10^5^ CFU/mL of unstained *Klebsiella* sp. was extremely weak, and the fluorescence was mainly biological autofluorescence. As shown in Figure 9b, the relative fluorescence intensity of *Klebsiella* sp. labeled with wh-CQDs in the P2 region was significantly enhanced, which could be distinguished from the unstained cell clusters. The P2 region incubated for 12 h accounted for 32.89% of the total cell number, indicating that the proportion of wh-CQDs-labeled *Klebsiella* sp. accounted for 32.89% of the total cell number. Similarly, in Figure 9c, the proportion of labeling accounted for 41.78% of the total cell number incubated for 24 h. Therefore, these data highlight a different time course and proportion of internalization of wh-CQDs in *Klebsiella* sp. cells.

## 4. Conclusions

In the present study, blue fluorescent CQDs were derived from natural biomass wh using a bottom-up hydrothermal method at 180 °C for 12 h. The synthesized wh-CQDs were uniform in size, with an average size of 2.44 ± 0.57 nm, and exhibited typical excitation-light-dependent properties owing to surface state defects. The wh-CQDs exhibited excellent physicochemical properties with high selectivity and sensitivity to Fe^3+^. In addition, toxicity and imaging studies on the hydrogen-producing bacterium *Klebsiella* sp. and flow cytometry analysis of intracellular wh-CQDs were performed for the first time using biomass-derived carbon dots. Owing to the green synthesis of wh-CQDs, even at high concentrations (1000 μg/mL), wh-CQDs showed low cytotoxicity toward hydrogen-producing bacteria. Notably, the synthesized wh-CQDs possessed strong penetrating ability and good biocompatibility, and were able to break through the lipid and polysaccharide barrier in *Klebsiella* sp. cell wall. The wh-CQDs entered bacterial cells and emitted bright blue fluorescence, implying that they may be an effective probe for flow cytometry. However, the long-term performance of wh-CQDs tracer analysis in *Klebsiella* sp. is still lacking, and the analysis of bacterial cell imaging mechanism is not deep enough, which needs to be further improved in the future research work. Overall, our study expands the application of biomass carbon spots and demonstrates that wh carbon spots can be used as sensitive fluorescent probes for Fe^3+^ detection and cellular imaging.

## Figures and Tables

**Table 1:** Comparison of the Fe^3+^ detection sensitivity between the wh-CQDs and other reported CQDs.

Sensing Platform	Carbon Source	Synthesis Method	Linear Range (μM)	LOD (μM)	R^2^	Ref.
Nitrogen-doped carbon dots	Poa pratensis	Carbonization	5–25	1.4	0.997	[47]
C-dots	Ananas erectifolius	Hydrothermal	0–30	0.77	0.997	[1]
CDs	Mint	Carbonization	0–400	0.037	0.995	[58]
S-doped carbon quantum	Ascorbic acid and thioglycolic	Hydrothermal	0–200	0.05	0.995	[59]
N-CDs	Diethylenetriamine	Hydrothermal	2–50	10.42	0.996	[60]
Wh-CQDs	Water hyacinth	Hydrothermal	0–330	0.084	0.996	This work

## Data Availability

The data will made available on request.

## Supplementary Materials

The following are available online at https://www.mdpi.com/article/10.3390/nano12091528/s1, Table S1. Wh-CQDs and Quinine Sulfate Concentration for Measuring QY; Figure S1. Selectivity of the wh-CQDs toward different metal ions.
