# Supplementary material for: Facile and Green Synthesis of Highly Fluorescent Carbon Quantum Dots from Water Hyacinth for the Detection of Ferric Iron and Cellular Imaging
Source: Nanomaterials (Basel). 2022 May 1;12(9):1528. doi: 10.3390/nano12091528 (PMC9100092; doi:10.3390/nano12091528)
Supplement: Supplementary file 1 [file nanomaterials-12-01528-s001.zip › nanomaterials-1691433-supplementary.pdf]

nanomaterials

MDPI

*Supplementary Materials*

# Facile and Green Synthesis of Highly Fluorescent Carbon Quantum Dots from Water Hyacinth for the Detection of Ferric Iron and Cellular Imaging

**Pei Zhao [1], Qin Zhang [1,2,*], Juanjuan Cao [1], Cheng Qian [1], Jing Ye [1], Siyuan Xu [1], Yonggui Zhang [1] and Yanbin Li [1,2,*]**

[1] College of Biological and Food Engineering, Anhui Polytechnic University, Wuhu 241000, China; lvxmzp@163.com (P.Z.); cjj18890973009@163.com (J.C.); qiancheng9981@163.com (C.Q.); a2235672022@163.com (J.Y.); shanyazi0605@163.com (S.X.); xjagcdzyg@163.com (Y.Z.)

[2] Anhui Engineering Laboratory for Industrial Microbiology Molecular Breeding, College Biological and Food Engineering, Anhui Polytechnic University, Wuhu 241000, China

* Correspondence: jhtabszq@163.com (Q.Z.), lyb@ahpu.edu.cn (Y.L.)

**Table S1.** Wh-CQDs and Quinine Sulfate Concentration for Measuring QY.

| Number | Concentration of Quinine Sulfate (μg/mL) | Absorbance | Concentration of Wh-CQDs (μg/mL) | Absorbance |
|---|---|---|---|---|
| 1 | 1 | 0.016 | 3.17 | 0.036 |
| 2 | 2 | 0.032 | 6.36 | 0.048 |
| 3 | 3 | 0.043 | 9.22 | 0.062 |
| 4 | 4 | 0.057 | 12.41 | 0.079 |
| 5 | 5 | 0.074 | 15.53 | 0.092 |

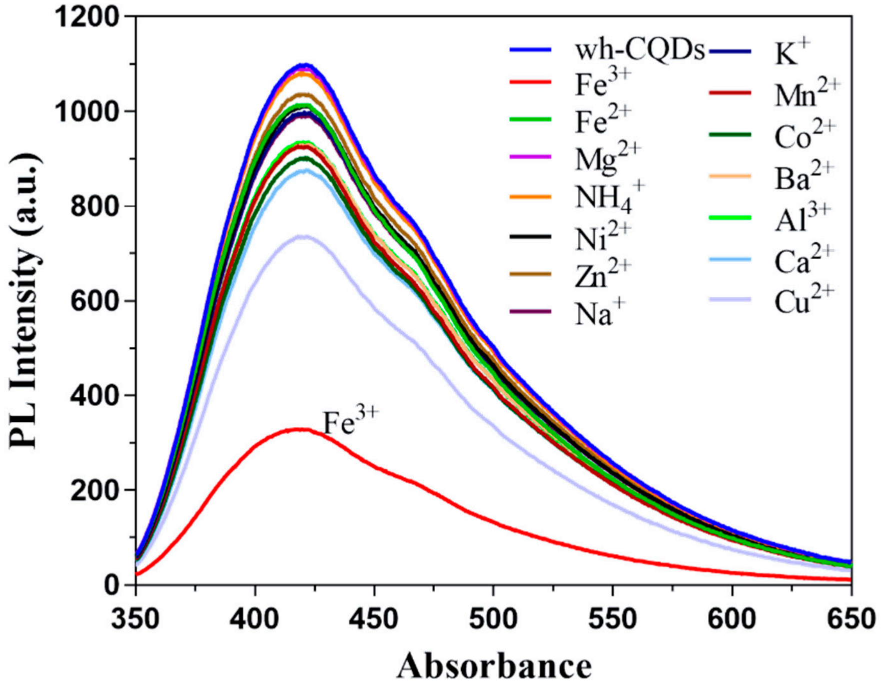


**Figure S1.** Selectivity of the wh-CQDs toward different metal ions.
